# Identification and Characterisation of a Hyper-Variable Apoplastic Effector Gene Family of the Potato Cyst Nematodes

**DOI:** 10.1371/journal.ppat.1004391

**Published:** 2014-09-25

**Authors:** Sebastian Eves-van den Akker, Catherine J. Lilley, John T. Jones, Peter E. Urwin

**Affiliations:** 1 Centre for Plant Sciences, University of Leeds, Leeds, United Kingdom; 2 Cell and Molecular Sciences Group, Dundee Effector Consortium, James Hutton Institute, Invergowrie, Dundee, United Kingdom; Rush University Medical Center, United States of America

## Abstract

Sedentary endoparasitic nematodes are obligate biotrophs that modify host root tissues, using a suite of effector proteins to create and maintain a feeding site that is their sole source of nutrition. Using assumptions about the characteristics of genes involved in plant-nematode biotrophic interactions to inform the identification strategy, we provide a description and characterisation of a novel group of hyper-variable extracellular effectors termed HYP, from the potato cyst nematode *Globodera pallida*. HYP effectors comprise a large gene family, with a modular structure, and have unparalleled diversity between individuals of the same population: no two nematodes tested had the same genetic complement of HYP effectors. Individuals vary in the number, size, and type of effector subfamilies. HYP effectors are expressed throughout the biotrophic stages in large secretory cells associated with the amphids of parasitic stage nematodes as confirmed by *in situ* hybridisation. The encoded proteins are secreted into the host roots where they are detectable by immunochemistry in the apoplasm, between the anterior end of the nematode and the feeding site. We have identified HYP effectors in three genera of plant parasitic nematodes capable of infecting a broad range of mono- and dicotyledon crop species. *In planta* RNAi targeted to all members of the effector family causes a reduction in successful parasitism.

## Author Summary

Sedentary plant parasitic nematodes are pathogens that invade plant roots and establish a feeding site. The feeding site is a specialist structure used by the nematode to support its development within the plant. The nematode secretes a suite of proteins, termed ‘effector proteins’ that are responsible for initiating and maintaining the feeding site. The nematode must also evade recognition by the plant defence systems throughout its lifecycle that can last for many weeks. We describe a diverse and variable effector gene family (HYP), the products of which are secreted into the plant by the nematode and are required for successful infection. The variability and modular structure of this gene family can lead to the production of a large array of effector proteins. This diversity may allow the nematodes to combat any resistance mechanisms developed by the plant. Each nematode tested within a population is genetically unique in terms of these effector genes. We found huge variation in the number, size and type of HYP effectors at the level of the individual. This may explain some of the difficulties in breeding nematode resistant plants and has profound implications for those working with other plant pathogens.

## Introduction

Plant parasitism by nematodes is a major threat to global food security, with at least one nematode species targeting each of the world's most economically important crops [1]. Damage to crops caused by plant-parasitic nematodes worldwide has been valued at over £75 billion each year [2]. A better understanding of the mechanisms by which these organisms parasitise plants has the potential to make a significant contribution to global food security. Plant parasitic nematodes display a wide range of parasitic strategies, from simple migratory ectoparasites that live in soil and feed on root epidermal cells, to migratory endoparasites that feed destructively as they move through roots. However, the most complex, well-adapted, economically important, and consequently most widely studied are the sedentary endoparasites, including the root-knot and cyst nematodes of Clade 12 of the phylum Nematoda [3]. These biotrophic pathogens invade the host roots as second stage juveniles (J2) and migrate to cells near the vascular cylinder. A suite of “effector proteins”, which modify host tissues to create a feeding site, are injected into root cells via a needle-like stylet. The female nematodes will feed from these sites for a period of 4–6 weeks while they develop and swell into mature, egg producing adults [4]. At the time of induction of the feeding site the nematode becomes sedentary, losing the ability to move. If at any time during these 4–6 weeks the feeding site is compromised the nematode cannot survive. Nematodes, like other biotrophic plant pathogens, have therefore evolved the ability to suppress host defences (reviewed in [5], [6]).

The ability to manipulate host processes and induce complex feeding sites appears to have evolved independently in the root-knot and cyst nematodes [3]. Root-knot nematodes induce the formation of giant cells while cyst nematodes induce syncytia. Although giant cells and syncytia show similar cellular features including reduced vacuoles and extensive proliferation of the smooth endoplasmic reticulum, ribosomes, mitochondria and plastids [7], [8], their development and ontogeny are entirely different. Root-knot nematodes induce multiple rounds of mitosis in the absence of cytokinesis to generate the multinucleate giant cell. Cyst nematodes, on the other hand, promote dissolution of cell walls and protoplast fusion of hundreds of adjacent cells to generate the syncytium [8].

Much recent work for both cyst and root-knot nematodes has focused on identification of effectors and their localisation within the host tissue. It is becoming increasingly clear that the apoplasm is an important recipient compartment for effectors during both the migratory and sedentary stages. The CLAVATA-like or CLE peptides of *Heterodera schachtii* are expressed in the dorsal pharyngeal gland cell throughout infection [9], and are apparently secreted into syncytia then subsequently transported to the apoplasm by existing plant mechanisms [10]. The MAP-1 protein of *Meloidogyne incognita*, initially identified as a putative avirulence factor is secreted by the amphids [11] and accumulates in the apoplasm [12], [13]. Similarly the Mi-ASP2 and Mi-PEL3 proteins are secreted into the apoplasm by the subventral glands during both migration of the juvenile and the sedentary feeding stages of mature females [12]. In addition, electron microscopy studies of feeding cyst nematodes have identified a structure produced by endoparasitic nematodes that is directly associated with the plant-nematode interface; the feeding plug [14], [15]. Feeding plugs appear as electron dense material located in the apoplasm between the anterior end of the nematode and the feeding site [14].

Until recently, nematode effector identification was centred on the dorsal or subventral pharyngeal gland cells, either by localisation of sequenced gene expression to these structures or by direct isolation of RNA from the pharyngeal gland cells [16], [17]. These have successfully identified various effectors with a range of functions [5], [6] however these strategies may fail to identify effectors involved in maintenance and suppression of host defences throughout the biotrophic phases, in particular those that do not originate from the gland cells. Indeed, nematodes have numerous other tissues with the capacity to secrete proteins into their host. The amphids are the primary sense organs and their function was thought to be confined to the migratory stages of sedentary endoparasitic nematodes (reviewed in [18]). However, structural changes occur in the amphids during the transition from migratory juvenile to sedentary feeding stages [19], suggesting distinct roles at each stage. Two previous studies showed the feeding plugs of cyst nematodes are continuous with the amphid openings of sedentary females [14], [15], one of which concluded that the feeding plug originates from the amphidial canal [14]. In *Meloidogyne* species, MAP-1 proteins are secreted into the host from the amphid opening [12], [20]. Moreover, a functional glutathione peroxidase is secreted from the hypodermis of *G. rostochiensis*, and may play a role in breaking down host reactive oxygen species during infection [21]. Finally, a Cellulose Binding Module2-bearing protein accumulates near the vagina of gravid female *M. incognita* [12] and may originate from the rectal glands.

We therefore hypothesise there would be a class of effectors, crucial to the successful biotrophic interaction, that would require continual renewal throughout biotrophy and may not originate from the gland cells. The advent of next generation sequencing, in particular RNA sequencing, has provided new approaches to many questions in biology. Here we describe the use of RNAseq data to identify and characterise effectors with continual expression throughout the biotrophic phases of cyst nematodes. We present the first description and characterisation of a hyper-variable extracellular effector gene family, termed HYP effectors.

## Results

**Figure 1:**
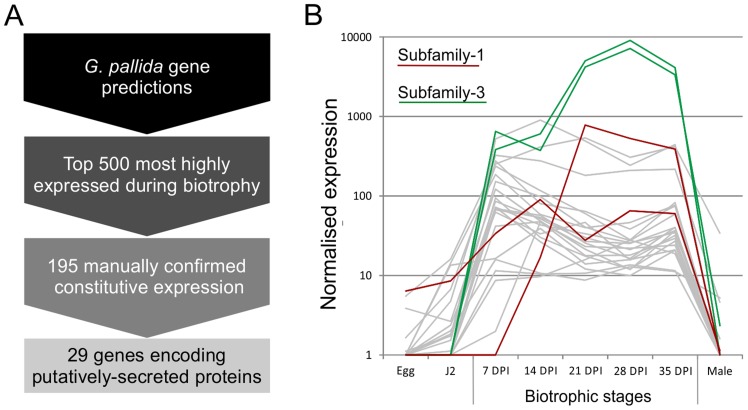
Pipeline for identification of genes involved in plant-nematode biotrophy. A) Summarises the process of identifying novel genes, highly up regulated during biotrophy, that encode secreted proteins. B) Example expression data of all candidates with the addition of all other full length or partial HYP effector sequences present in the genome assembly, highlighted in red and green for subfamily-1 and -3 respectively.

Using the assumption that effectors involved throughout the biotrophic interaction would be specifically and abundantly expressed in all sedentary stages, and would encode secreted proteins, a pipeline was developed to identify the most promising candidate genes (Figure 1). All predicted genes in the recently assembled genome sequence of the potato cyst nematode *Globodera pallida* [22] were ranked by their ratio of normalised expression during biotrophic stages compared to non-biotrophic stages. The top 2% (≈500) of genes were manually verified, and those that were not evenly highly expressed across all biotrophic stages were discarded. The remaining 195 sequences were taken forward for secreted protein prediction. This resulted in 56 proteins with predicted signal peptides, 29 of which had no transmembrane domains. These 29 genes were compared to all published sequences in the non-redundant database, and those that had no significant homologues or no conserved domains were analysed further. One gene family identified, subsequently named HYP, was characterised in detail.

### *Gp-hyp* gene family identification and cloning

Based on the genomic sequence of GPLIN_001208400 (ftp://ftp.sanger.ac.uk/pub/pathogens/Globodera/pallida/Gene_Predictions/), primers were designed to amplify the coding region after the predicted cleavage site of the signal peptide to the stop codon (Table S1). This primer pair amplified a range of different sized products, which when sequenced could be grouped into three subfamilies -1 -2 and -3, each sharing considerable stretches of conserved bases at the 5′ and 3′ ends of the genes. Each subfamily was compared back to the genome by BLAST. Two complete genes were present, the previously identified GPLIN_001208400 and GPLIN_001025300, corresponding to subfamily -1 and -3 respectively. There were also two gene fragments (either a partial sequence or containing poly-N regions), named GPLIN_001135100 and GPLIN_000907700, corresponding to subfamilies -1 and -3 respectively. Based on the genomic sequence of gene GPLIN_001208400, primers were designed to amplify from the start to the stop codon. These specifically amplified a range of different sized products that could be grouped into subfamily -1 and -3 only.

**Figure 2:**
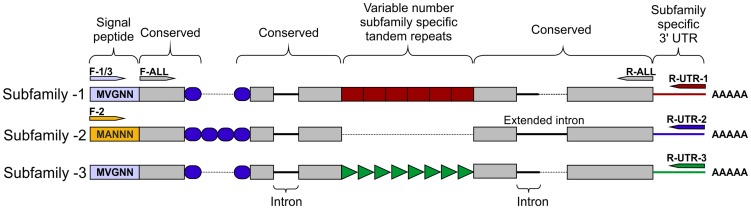
HYP effector gene family schematic and location of primer combinations. All HYP effector genes identified to date encode a secretion signal followed by two highly conserved regions flanking a region of variable number tandem repeats, and a subfamily specific 3′ UTR. Conserved regions of *Gp-hyp* genes share >95% identity between subfamilies. Subfamily-2 has a slightly different signal peptide (highlighted). 3′ UTRs are identical within subfamilies, irrespective of number of tandem repeats. Members of all subfamilies can be amplified using the F-ALL and R-ALL primer pair, subfamilies can be distinguished by PCR using a combination of signal peptide and 3′ UTR primers (summarised in Table S1). Solid lines represent introns, while dashed lines represent alignments.

No subfamily-2 members were present in the assembled genome sequence, however two *Gp-hyp* sequences were present in the *de novo* transcriptome assembly of early sedentary stage nematodes (7 days post infection), both of which corresponded to subfamily -2. Primers designed to amplify from the start to the stop codon amplified a range of products that all corresponded to subfamily -2. Subfamilies -1 and -3 were not separable by PCR of coding regions. 3′ RACE was carried out for each of the three subfamilies, which identified a single specific UTR per subfamily (Figure 2). Each single subfamily specific 3′ UTR corresponded to the expected 3′ UTR sequence present for that subfamily from either the assembled genome or *de novo* transcriptome.

### Inter-subfamily variation

Subfamily-specific PCR primers from start codon to 3′ UTR (Figure 2) were able to differentiate between subfamilies. In general subfamilies and locations of primer combinations can be described by Figure 2. In total, 75 unique genomic sequences were identified across *Gp-hyp-1*, -*2* and -*3*, where *Gp-hyp-1* is numerically dominant. All cloned *Gp-hyp* genes, irrespective of subfamily, shared stretches of 410 and 94 nucleotides with >90% identity at the 5′ and 3′ ends respectively. Between highly conserved regions, subfamilies are characterised by a series of variable number subfamily-specific tandem repeats, summarised in Figure 2. No *Gp-hyp* sequences identified to date encode any annotated domains with the exception of a predicted signal peptide at the N-terminus of the protein.

### Intra-subfamily variation

**Figure 3:**
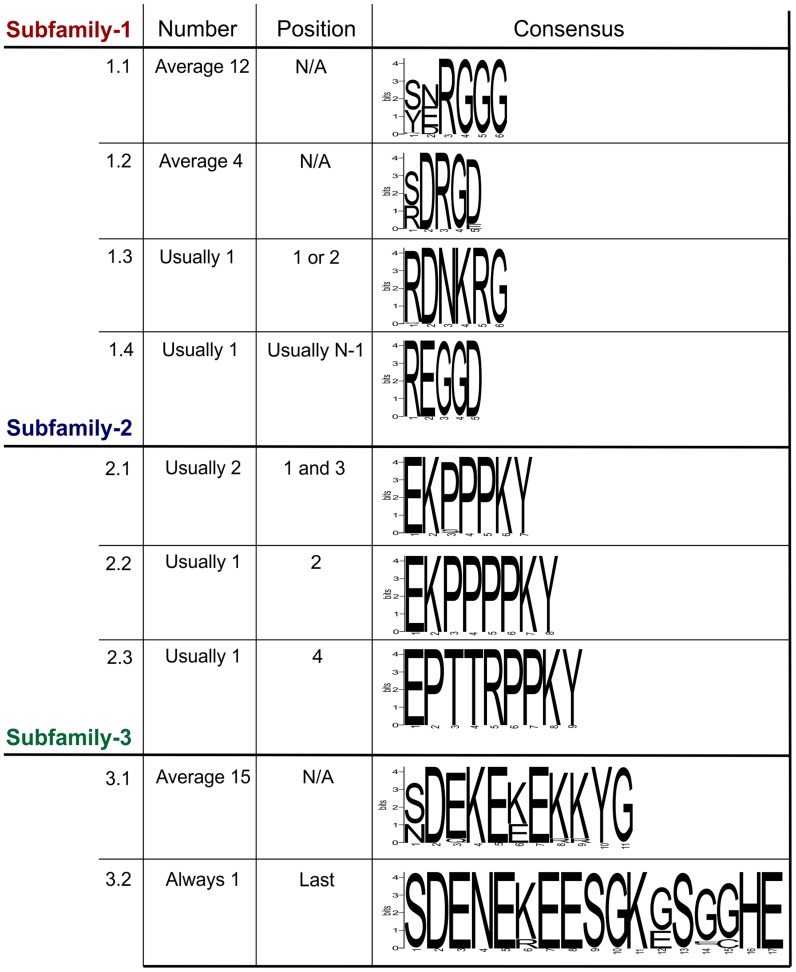
Summary of the variation within the tandem repeat domains. Deduced amino acid sequences from the tandem repeat regions of genomic DNA sequences are shown. For each subfamily, the motifs present in the tandem repeat domain are shown along with their average copy number, position and consensus sequence. For *Gp-hyp*-1 and -3 no pattern has emerged to the positions/organisations of the highest frequency motifs. Motif 1.1 has the greatest variation in tandem repeat number, although motif 3.1 has less variation in the number of tandem repeats it has the greatest variation in the motif consensus sequence.

Within each subfamily multiple different genomic DNA sequences were amplified, cloned and sequenced. In all cases, the entire tandem repeat region consists of a single open reading frame. For *Gp-hyp*-*1* and -3 only, large variation is observed in the number, sequence and order of tandem repeats in the deduced amino acid sequences corresponding to different genomic sequences. Within this region, *Gp-hyp*-1 genes contain four motifs, two of which are present as tandem repeats with complex organisations (Figure 3). The most common motif (1.1) consists of 6 amino acids, the first two are variable followed by a highly conserved RGGG. This glycine rich motif is present on average 12 times per gene, although this varies greatly. The second motif (1.2) consists of 5 amino acids, with a variable first position followed by conserved DRGD. This motif is present approximately 4 times per gene. The other two motifs (1.3 and 1.4) are usually present no more than once, often at the start or end of the tandem repeat domain (Table S2). Including all variable regions of all motifs in subfamily-1, 20 unique amino acid sequences are encoded by 43 unique nucleotide sequences.

The *Gp-hyp*-2 tandem repeat region always contains four proline rich tandem repeats, comprised of three different motifs (2.1, 2.2 and 2.3). Motif 2.1 consists of 7 amino acids in the sequence EKPPPKY. Motif 2.2 is identical, except for the inclusion of an additional proline in the proline rich repeat (EKPPPPKY) and has no variation in sequence. Motif 2.3 consists of 9 amino acids, also has no sequence variation and is usually present only once (Figure 3). If motif 2.1 is present in positions 1 and 3, the position 3 motifs are more similar at the nucleotide level to other 2.1 motifs in position 3, than they are to 2.1 motifs in position 1, even if they are identical at the amino acid level. The four subfamily-2 tandem repeat variants are encoded by 10 unique nucleotide sequences. Interestingly, both subfamily-1 and -3 also contain the first and last of this type of tandem repeat.

*Gp-hyp*-3 tandem repeat regions contain two lysine and glutamic acid rich motifs (3.1 and 3.2) the first of which (3.1) occurs in tandem repeats on average 15 times, although this varies greatly. Motif 3.1 consists of 11 amino acids with highly conserved amino acids in position 2, 4, 5, 7, 10 and 11. Motif 3.2 consists of 17 amino acids and is always present as a single copy in the final position of the tandem repeat domain of subfamily-3 (Figure 3 and Table S2). Including all variable regions of all motifs in subfamily-3, 27 unique amino acid sequences are encoded by 42 unique nucleotide sequences.

Organisation of the various motifs within the tandem repeat region of all subfamilies is summarised in Table S2, although no pattern can describe all the variation. The above analysis was carried out using amino acid sequences deduced from genomic DNA sequences. Representatives of all subfamilies are present in cDNA synthesised from mRNA extracted from feeding females and these display similarly variable numbers and arrangements of tandem repeats. However, these are not studied in any detail due to the introduction of non-canonical apparent splice events as a direct result of the reverse transcriptase enzyme activity (described in more detail in Supporting information S2 and Figure S3).

**Figure 4:**
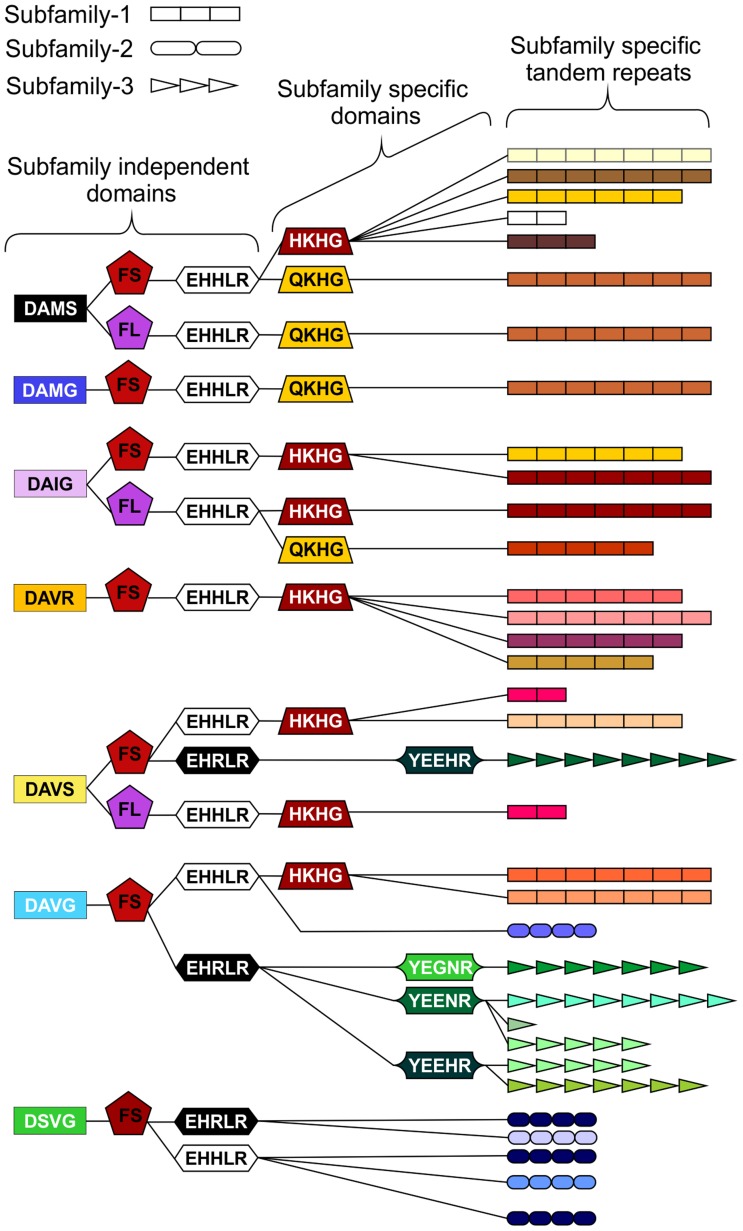
Schematic representation of HYP effector domain organisation in *G. pallida*. Within the conserved regions, non-synonymous SNPs result in various domains. Combinations of domains give rise to “types”. Certain types are subfamily specific, whereas others are not. For each column only, the same colour indicates the same sequence. By organising the sequences using types as indicators (not in the order they appear in the genes), HYP effectors do not group by subfamily. The three different subfamily specific tandem repeats are indicated in the final column as different shapes, where the size roughly correlates to number of repeats. Sequences encoding similar tandem repeat regions can have different domains at every locus, and similarly sequences encoding identical domains at every locus can have different tandem repeat regions.

In addition to the variable tandem repeats, within the highly conserved regions several non-synonymous polymorphic sequences have been identified that lead to amino acid changes. Figure 4 shows a schematic representation of these domains and does not represent the order in which they appear in the genes. Each combination of domains can be referred to as a “type”. Types are not subfamily specific. Moreover, sequences encoding similar tandem repeat regions can have different amino acid sequence at every domain locus, and similarly identical types at every domain locus can have different tandem repeat regions (Figure 4 and Table S2).

### Copy number variation between individuals

**Figure 5:**
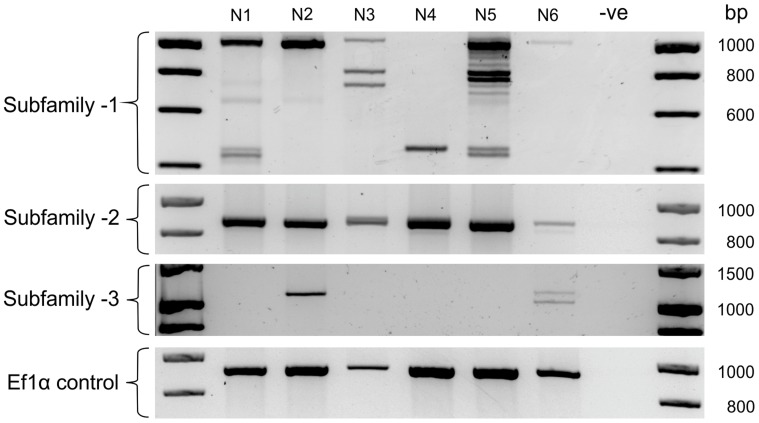
HYP effector variation between individuals. Each lane shows PCR products amplified from discrete nematode samples. Each PCR in lane one corresponds to DNA extracted from the same single *G. pallida* nematode. PCR on 6 individual nematodes using subfamily specific primers highlights a profound difference between individuals. In particular for subfamily-1 and -3 a reproducible difference can be seen in the complement of HYP effectors between nematodes both within and between subfamilies. The identity of amplification products from various samples was confirmed by sequencing. No two nematodes tested had the same genetic complement of HYP effectors.

The large number of unique *Gp-hyp* genomic DNA sequences were cloned from a pool of thousands of individual nematodes. To determine the complement of *Gp-hyp* sequences present in each nematode, a method was developed to extract high quality DNA, sufficient for multiple PCR reactions, from individual feeding female cyst nematodes. PCR with *Gp-hyp* subfamily-specific primers (*Gp-hyp*-1 F-1/3 and R-UTR-1; *Gp-hyp*-2 F-2 and R-UTR-2; *Gp-hyp*-3 F-1/3 and R-UTR-3; summarised in Table S1) revealed that individuals within a population differ in the number of *Gp-hyp* sequences, the length of *Gp-hyp* sequences, and in some cases even the presence or absence of *Gp-hyp*-*3* genes. No two nematodes tested had the same complement of HYP effectors (Figure 5). Amplicons from individual PCR reactions were sequenced and this confirmed that a single nematode can contain more than one type, even within subfamilies. These data indicate a profound difference between individuals at the genetic level.

### HYP effectors are expressed in large secretory cells associated with the amphids

**Figure 6:**
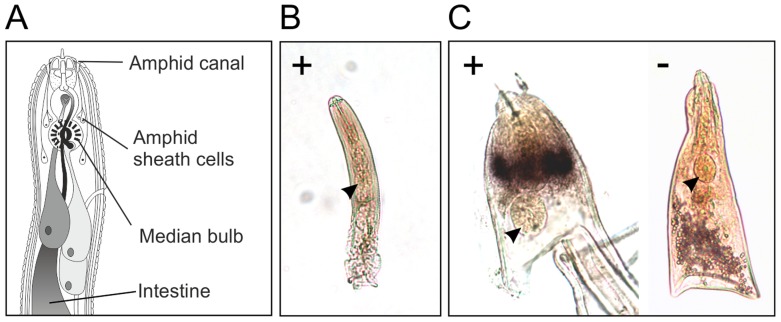
HYP effectors are expressed in the amphid sheath cells of parasitic females. A) Schematic representation of *Globodera pallida* feeding female [64]. *In situ* hybridisation probe designed to a conserved region common to all HYP effectors identifies extremely strong and specific dark staining in a paired structure anterior to the metacorpal bulb of 14 days post infection females (C). No such staining pattern is observed with the negative control, or the positive control on second stage juvenile (J2) nematodes (B). Comparison of the staining pattern with a schematic identified this structure as the two lobed amphid sheath cells.

*In situ* hybridisation was used to determine the spatial expression patterns of *Gp-hyp* genes in feeding *G. pallida* females. A probe designed to target a conserved region of all subfamilies localised transcripts to a paired structure anterior to the metacorpal pump chamber. No such staining pattern was seen using the negative control probe (Figure 6). When compared to the anatomy of a typical tylenchid nematode, the position of the signal is consistent with expression in the amphidial sheath cells. No other paired structures are present in this region of the nematode. Amphid sheath cells are large secretory cells that produce material present in the amphidial canal and at the anterior surface of the nematode [14]. No expression was observed in pre-parasitic J2 nematodes (Figure 6), as expected from the highly significant up-regulation of HYP effectors specifically in all biotrophic stages (p-values range from 1.68*10^−3^ to 5.08*10^−9^ depending on the genomic copy; Figure S1).

### Gp-HYP proteins are secreted by the nematode and accumulate at the host-parasite interface

**Figure 7:**
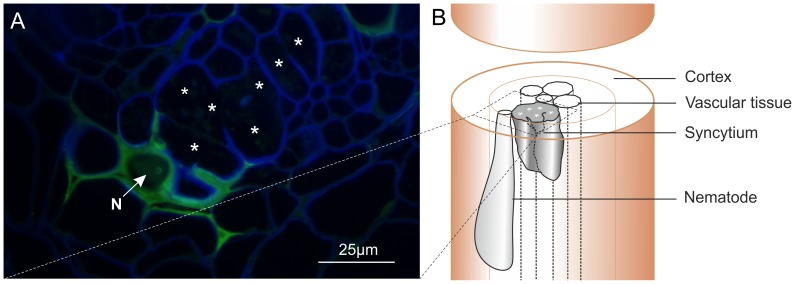
Immunolocalisation of Gp-HYP proteins to the apoplasm. A) 2 µm section showing Gp-HYP protein (green) detected between cell walls (blue) in the apoplasm between the feeding site (*) and the anterior end of the nematode (N). Cell walls are stained with calcofluor white (blue). B) Comparison with a schematic highlights the nematode in the context of the syncytium and the root tissue.

Polyclonal rabbit antibodies were raised against a synthetic peptide derived from Gp-HYP sequences. Anti-Gp-HYP antibody was able to detect several proteins in a pooled nematode extraction and did not detect a signal when using similar quantities of plant root protein tested by western blot (Figure S2 B). Pre-immune serum was unable to detect the same proteins (Figure S3 B). Anti-Gp-HYP antibody specifically detected recombinant Gp-HYP-2 protein expressed and purified from bacteria, but not an unrelated negative control candidate effector protein similarly produced (Figure S3 A). The anti-Gp-HYP antibody localised a protein present in the apoplasm 14 days post infection at the plant-nematode interface, between the nematode and the syncytial cell wall (Figure 7).

### *In planta* RNAi targeting HYP effectors causes a reduction in successful plant-parasitism

**Figure 8:**
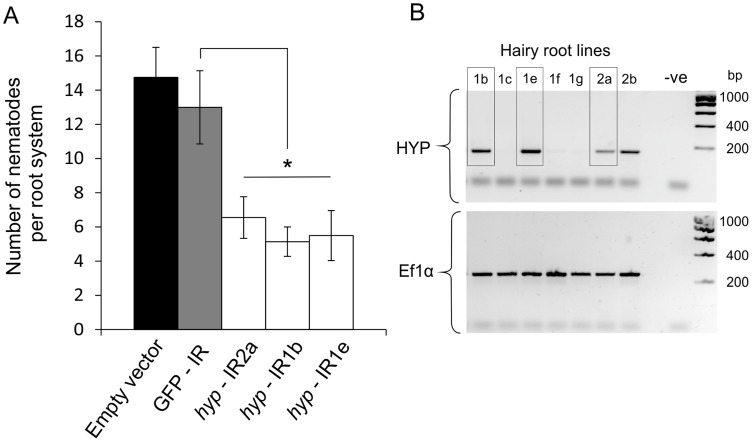
RNA interference of *Gp-hyp* genes. A) An inverted repeat construct targeting a single *Gp-hyp* 1 gene was transformed into hairy roots of potato. Due to the nature of the conserved regions the construct was expected to target all *Gp-hyp* genes in all subfamilies. For all *Gp-hyp*-IR lines a significant reduction in nematode numbers (p<0.05) was seen when compared to the GFP IR hairy roots (n = 8). Error bars indicate the standard error of the mean. B) Semi-quantitative RT-PCR confirms expression of the inverted repeat construct in the RNAi lines. Line 2b was not tested for resistance as the phenotype varied too greatly from the GFP and empty vector controls.

A representative *Gp-hyp-1* cDNA sequence was used to create an inverted repeat (IR) construct to express double stranded RNA *in planta* under the control of the CaMV35S promoter. As a result of the highly conserved regions, this construct should target all *Gp-hyp-1*, -*2* and -*3* transcripts. RT-PCR was used to confirm expression of the inverted repeat constructs *in planta* (Figure 8B). All potato hairy root lines tested that expressed the *Gp-hyp-1* hairpin construct resulted in a 50–60% reduction in the number of nematodes that successfully infected and induced a feeding site compared to GFP IR control (correcting for multiple T-tests p<0.05), suggesting a role of HYP effectors during parasitism (Figure 8A). Hairy root line 2b was not tested as its growth phenotype was not comparable to the control roots.

### HYP effectors are specific to obligate biotrophic plant-parasitic nematodes

**Figure 9:**
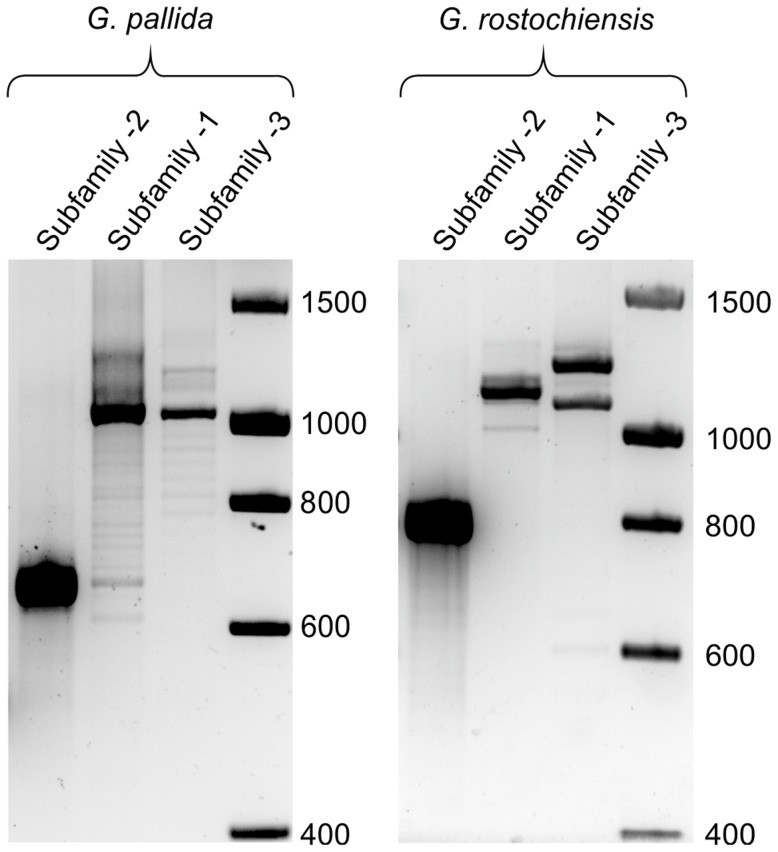
Comparison of HYP effector variation between UK populations of *G. pallida* and *G. rostochiensis*. For *Gp-hyp* subfamily -1 and -3 large variation can be seen when amplifying from DNA extracted from a pool of thousands of *G. pallida* as indicated by multiple bands. Considerably less variation is seen under the same reaction conditions for *G. rostochiensis*, probably reflecting a more genetically restricted introduction to the UK. Amplicons from each reaction were sequenced to confirm they correspond to HYP effectors.

**Figure 10:**
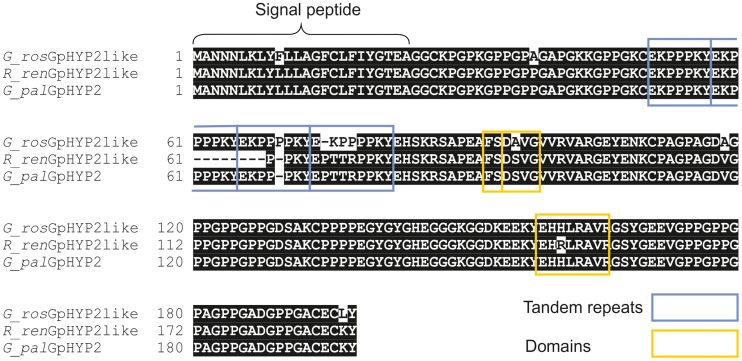
Conservation of *hyp* genes in *Globodera* and *Rotylenchulus* species. Amino acid alignment of HYP-2 sequences from *G. pallida, G. rostochiensis* and *R. reniformis*. HYP-2 sequences are highly conserved (>93%) across the full length of the protein in the three species. The tandem repeat region, and true locations of the subfamily independent domains are highlighted.

We used bioinformatic analysis to investigate the distribution of the HYP effectors in a variety of plant-parasitic nematodes. The genome assembly of the closely related yellow potato cyst nematode *Globodera rostochiensis* (unpublished) contained *Gp-hyp-1*, *-2* and *-3* like sequences. Due to the nature of HYP effectors and the apparent difficulty in assembly, their presence in *G. rostochiensis* was confirmed by PCR (primers *Gp-hyp*-1 F-1/3 and R-UTR-1; *Gp-hyp*-2 F-2 and R-UTR-2; *Gp-hyp*-3 F-1/3 and R-UTR-3; summarised in Table S1). Amplification products from *G. rostochiensis* were sequenced to confirm they did indeed correspond to HYP effectors. However, notably fewer HYP effectors are present in *G. rostochiensis* compared to G. *pallida* (Figure 9). *Gp-hyp-1*, *-2* and *-3* like sequences were present in the genome sequence of the more distantly related cyst nematode *Heterodera glycines* (patent WO 2007095469). Two partial *Gp-hyp-1*-like and one *Gp-hyp-2*-like sequences were also present in the transcriptome of *Rotylenchulus reniformis* (unpublished and [23]). No HYP effector-like sequences were identifiable from the EST database of the migratory endoparasite *Radopholus similis* [24] or the genome sequence of the root-knot nematodes *Meloidogyne incognita* [25] or *Meloidogyne hapla* [26]. Interestingly the *Gp-hyp*–like sequences from the various nematode species described show a remarkable level of conservation between species and indeed between genera (Figure 10).

## Discussion

For this study we made several assumptions about the characteristics of effectors important throughout the biotrophic interaction of sedentary endoparasitic nematodes. These effectors are expected to be expressed throughout the parasitic stages of the nematode life cycle, and to be absent from all other stages. They are expected to be expressed in a tissue with the capacity to secrete proteins, with no prejudice for the pharyngeal gland cells. The genes are expected to encode proteins that are secreted and which should be detectable inside the host. By following this series of assumptions we have identified the HYP effectors: an incredibly complex and highly conserved gene family present across at least three genera of plant parasitic nematodes infecting mono- and dicotyledons.

### The role of amphids during the feeding stages of *Globodera pallida*

Until recently, nematode effector identification was centred on the dorsal or subventral gland cells. We exploited the highly conserved structure of HYP effectors to design an *in situ* hybridisation probe that would target all members of all subfamilies, irrespective of the individual genetic complement of *hyp* genes each nematode had. We were able to demonstrate expression in the amphid sheath cells, large secretory cells associated with the amphids. Previous studies have shown that the cyst nematode feeding plug is continuous with the amphid openings of sedentary females [14], [15] and may originate from the amphidial canal [14]. Gp-HYP proteins are secreted from the amphids, however the proteins extend further in the apoplasm than expected when compared to electron micrographs of feeding plugs and so they are probably not structural components of the feeding plug. In *Meloidogyne* species the importance of the amphids and of the apoplasm as a site of effector action is now being recognised [12], [20]. The data presented here also suggest a changing role for the amphids in *G. pallida* throughout the life cycle. Interestingly, *map-1* genes of *M. incognita* also contain tandem repeats that differ between populations [27], although these have no sequence or structural similarity to HYP effector tandem repeat domains and are relatively simple in comparison. Recent data suggest these genes may in fact encode CLE-like peptides that originate from the pharyngeal glands [28]. HYP effector tandem repeats have no similarity to CLE peptides.

### HYP effectors are part of a complex multi-gene family

Despite the presence of just three complete *Gp-hyp* sequences across the assembled genome and transcriptome sequences of *G. pallida* (one corresponding to each subfamily), the complexity of the HYP effector family was identified by conventional PCR and sequencing of individual clones. In addition, each time more clones were sequenced more unique sequences were identified. This suggests that the 75 unique genomic sequences identified represent a far from exhaustive list, and that the full complexity of HYP effectors is yet to be catalogued. The absence of the full gene family in the *G. pallida* genome assembly, and the presence of two fragmented *Gp-hyp* genes in poly-N regions, highlights a limitation of sequencing and assembly of short reads. All *Gp-hyp* sequences, irrespective of subfamily, share stretches of 410 and 94 nucleotides with >90% identity at the 5′ and 3′ ends respectively which may underlie the difficulty in assembly.

### Comparison of HYP effectors to other pathosystems

All unique genomic HYP effector sequences identified to date can be readily assigned to one of three subfamilies primarily, although not only, based on the amino acid sequence of the tandem repeat region. Numbers of subfamily-specific tandem repeats range greatly. It is unclear what the function of the tandem repeats is in the context of plant parasitic nematodes. Variable tandem repeat Transcription Activator-Like (TAL) effectors have been described for *Xanthomonas spp* [29]. TAL effectors are highly adaptable phytobacterial virulence factors that contain a 34 amino acid tandem repeat present in 17.5 iterations. DNA-binding base pair specificity is conferred by variable di-residues in positions 12 and 13 within each tandem repeat. HYP effectors contain numerous shorter tandem repeats that are considerably more variable in both sequence and organisation. Tandem motif 1.1 does contain highly conserved regions directly preceded by variable di-residues. However, HYP effectors are localised in the apoplasm, and so DNA binding is unlikely, although other ligand binding may be possible. It is unclear what the role of the different subfamilies, variable number/organisation of tandem repeats, or domains is in the context of ligand binding. HYP effector tandem repeats of subfamilies -1, -2, and -3 contain conserved glycine, proline and lysine residues. Glycine residues often create flexible linkers between domains. Taken together, these data may allow suggest that variable residues interspersed by highly conserved linker regions may play a role in ligand binding.

Several non-synonymous SNPs were identified in HYP effectors, within the highly conserved regions, that do not group by subfamily. Various combinations of these domains are described here as “types”. Modularity of effectors has been demonstrated for the Crinkler and Necrosis (CRN) effectors of oomycete species [30], [31]. It is suggested that different domains in CRN effectors have discrete functions. The amino acid changes of HYP effector domains are usually physiochemically similar, and may be structurally superficial. The “type” structure of HYP effectors may reflect evolutionary origins and rearrangements rather than function, whereas the different subfamily tandem repeats, and the different number of tandem repeats within subfamilies, may reflect different functions. It has been noted however, that subtle amino acid changes that should conserve physiochemical properties can have an impact on effector function [32].

Similarly in oomycete species, the RXLR effectors are a large family of modular effectors with a highly conserved domain involved in membrane translocation [32]. HYP effectors are not characterised by a short conserved amino acid domain: stretches of hundreds of highly conserved amino acids are present at both the C and N-terminal ends of the protein. These highly conserved regions are not just conserved between species, but are conserved across at least three Genera of plant-parasitic nematodes.

### Copy number variation of HYP effectors between individuals of the same population

Despite the superficial genetic structural similarity of the HYP effector tandem repeats to TAL effectors, and the similar modularity compared to RXLR and CRN effectors, no effector families, from any pathosystem to date, have been identified with such complex variation between individuals of the same population. We have demonstrated un-paralleled genomic diversity of HYP effectors between individuals: no two nematodes tested had the same genetic complement of *Gp-hyp-1* or *-3* sequences. Nematodes differed in the length, number, and even presence/absence of entire gene subfamilies. It is unclear what the underlying genetic mechanism is that allows such variation between individuals. Due to the nature of the draft genome assembly of *G. pallida* we are unable to confirm that *Gp-hyp* sequences are paralogues, although the fact that individual nematodes differ in the size, and particularly number, of sequences within subfamilies suggests this is the case. The number and variation suggests that *Gp-hyp* genes are under high selection pressure. Gene expansions of cytochrome P450 genes have been described in *Anopheles* species [33], where estimates are of approximately 30 to 40 genes [34]. This is a good example as CYP450 genes, and in particular their copy number, have been linked to the extremely high selection pressure of resistance to DDT [34], [35], [36]. It is possible that the expansion of the HYP effector gene family in *G. pallida* may reflect a similarly high selection pressure from the host. Secreted components are the pathogen factors that are recognised by both pattern recognition receptors and resistance gene products. Diversity in *Gp-hyp* sequences may reflect the need to evade recognition in order to avoid detection. We have found that a UK population of *G. pallida* has considerably more variation in HYP effectors than *G. rostochiensis*. It has been suggested that the UK populations of *G. pallida* are from a broader genetic introduction than *G. rostochiensis*. If the diversity seen between individuals of *G. pallida* for the *Gp-hyp* genes is true on a wider genomic scale, this may explain the difficulty in identifying a broad spectrum resistance against *G. pallida* compared to *G. rostochiensis* [37]. Recent technological advances in sequencing the transcriptome/genome of single nematodes may identify wider differences between individuals [38].

The genome sequence of *G. pallida* was challenging to assemble [22]. This may also be due to the inherent genetic variation that we have demonstrated between individuals of the same population, even infecting the same plant. Genetic diversity in plant parasitic nematodes has never been studied on such a scale, the most relevant works being between (as opposed to within) populations [27], [39], [40], [41], [42]. Interestingly it has been suggested that genetic variability observed at the scale of a field or even of a region is already observed at the scale of a single plant within a field [40].

### HYP effectors are specific to, and play a role in, plant nematode biotrophic interactions

Transformation of cyst nematodes is not currently possible: In order to carry out gene knockout studies J2s can be soaked in double stranded RNA (dsRNA) or dsRNA can be delivered to feeding nematodes through *in planta* expression [43], [44]. RNA soaking was not attempted in this study as expression of the *Gp-hyp* genes is specific to all feeding stages (Figure S1). Instead, a hairpin construct was expressed in potato hairy roots. It has been reported that dsRNA expression in hairy roots is not ubiquitous and that phenotypes of the root cultures can vary, which may affect nematode infection [45], [46]. Phenotypes of empty vector control, GFP IR control, and *Gp-hyp* IR hairy root lines were therefore matched to the best of our ability before infecting with nematodes. Three independent lines expressing dsRNA targeting HYP effectors had reduced infection compared to the controls, suggesting HYP effectors play a role in the nematode infection. Despite the inherent noise of the hairy root system [45], RNAi targeting the *Gp-hyp* genes resulted in a significant reduction in nematode numbers. Although the dsRNA construct used has the potential to knock out all members of all subfamilies characterised to date, the list of cloned *Gp-hyp* sequences we have characterised is unlikely to be complete. Future work will focus on generating transgenic potato plants expressing the inverted repeat constructs of all three *Gp-hyp* gene subfamilies, potentially creating durable resistance across all cyst and reniform nematodes. Due to the patchy nature of hairy root transgene expression [45], [46], it is possible that successfully established nematodes had induced feeding sites from cells not expressing the dsRNA. It is therefore challenging to confirm the knockdown of *Gp-hyp* expression from the nematodes that successfully establish. Similarly due to the amplification step and transitive nature of RNAi in nematodes [47], subfamily-specific knockout studies are not possible.

Using bioinformatic analysis we have shown that HYP effectors are present in all cyst nematode species sampled and are also present in the closely related reniform nematode *R. reniformis*. Given the phylogenetic proximity of the reniform nematodes to the cyst nematodes [3], and the similarities in the feeding sites that they induce [48], it is likely that they share the same origin of sedentary parasitism. HYP effectors were not identifiable from the most closely related migratory plant parasitic nematode [49], *Radopholus similis* [24], which does not form specific biotrophic interactions [48]. Similarly, no HYP effectors were identified from *Meloidogyne* species [25], which have an independent origin of sedentary plant parasitism [3]. The high degree of similarity between all *Gp-hyp* genes, and in particular the similarity of 3′ UTRs within subfamilies, suggests that this is a very recent gene expansion. Presumably the high conservation in the coding region of the genes is linked to protein function. The function of the high degree of conservation in the 3′ UTR within subfamilies is unknown, although it may be related to regulation of gene expression [50], [51].

Using assumptions about the characteristics of genes involved in plant-nematode biotrophic interactions to inform identification strategy, we provide the first description and characterisation of the HYP effectors. HYP effectors may play an important role in plant-nematode interactions and represent a class of effectors with continual expression, and presumably continual function, throughout biotrophy. Future work will focus on elucidating the function/s and specificity of the various gene family members, variable number tandem repeats and various domain arrangements. There are many economically unimportant, and consequently poorly characterised, nematode species that induce syncytial feeding sites in their host roots [52], [53], [54], [55], [56]. HYP effectors appear to be specific to plant-nematode interactions involving syncytia; identifying HYP effectors in these species may provide an insight into a common underlying feature of a complex endo-parasitic relationship.

## Methods

### Nematode DNA extraction and molecular cloning

Genomic DNA was extracted from a pool of thousands of frozen J2 *G. pallida* (“Lindley”) or *G. rostochiensis* (Ro1) according to the protocol described by Cotton *et al.* [22]. PCR was routinely carried out using BioTaq Red DNA polymerase following the manufacturer's instructions for cycling conditions (Bioline) and the oligonucleotide primers and annealing temperatures listed in Table S1. PCR products were cloned by T-A cloning into the pGEM-T Easy vector (Promega) following the manufacturer's suggestions. Plasmid DNA was extracted from bacterial culture using a QIAprep Spin Miniprep Kit (Qiagen) following the manufacturer's instructions. Individual plasmids were sequenced at the service provided by Beckman Coulter Genomics.

### Single nematode nucleic acid extraction

Individual *G. pallida* females, collected 14 days post infection (dpi) of potato plants, were flash frozen in liquid nitrogen in individual 1.5 ml microfuge tubes. Nematodes were suspended in 200 µl “Chaos” buffer [57] (4.5 M guanidine thiocyanate, 2% N-lauryl sarcosine, 50 mM EDTA (pH 8.0), 0.1 M 2-mercaptoethanol, 0.2% antifoam-A) disrupted with a pipette tip, and lysed by vortexing. One volume of phenol∶chloroform∶isoamyl alcohol (25∶24∶1) was added to the sample, vortexed, and centrifuged at 10,000 *g* for 5 minutes. The upper aqueous phase was transferred to 1 volume of 70% ethanol with the addition of 20 ng carrier RNA (NucleoSpin RNA XS). Total nucleic acid was extracted from the sample using a NucleoSpin RNA XS column, and the NucleoSpin RNA/DNA Buffer Set following manufacturer's instructions. Two microliters of purified DNA was sufficient per 50 µl PCR reaction.

### 3′ Rapid Amplification of cDNA Ends (RACE)

RNA was extracted from 14 dpi feeding females of *G. pallida* using an RNeasy mini kit (Qiagen) following the manufacturer's instructions for animal tissues. 3′ RACE was carried out using the 5′/3′ RACE Kit (Roche) according to manufacturer's instructions. The supplied oligo dT primer was used to prime cDNA synthesis. Subfamily-specific forward primers GAGGTTATGACGAGCATCATC, GAAAGGGCGGAGACAAAG and TGAGCATCGTCTCCGTGCTG for subfamily -1, -2 and -3 respectively were identified by aligning all cloned sequences and visualising in an alignment viewer (BioEdit). The entire 3′ RACE PCR reaction was purified with a Qiaquick PCR purification kit (Qiagen) following the manufacturer's instructions. Purified amplification products were cloned by T-A cloning into the pGEM-T Easy vector (Promega) following the manufacturer's suggestions, and positive clones were confirmed by sequencing.

### *In situ* hybridisation

*In situ* hybridisation probes were designed to target a region of 134 conserved nucleotides at the 3′ end of the translated region of *Gp-hyp* transcripts using oligonucleotide forward (AACACGGAGGTTATGACGAG), and reverse (GCTTGCGAATGCAAATAT) primers respectively. Template was amplified from feeding nematode cDNA, and single stranded probes were synthesised from the template to incorporate digoxigenin labelled dUTP (Roche) using PCR by incubating at 94°C for 2 minutes followed by 35 cycles of 94°C for 15 seconds, 55°C annealing for 30 seconds, and 72°C extension for 90 seconds. Incorporation of DIG-labelled dUTP was confirmed by an apparent increase in size on agarose gel electrophoresis compared to template dsDNA. *In situ* hybridisation was carried out according to the methods described by de Boer *et al.* [58] with the following alterations. Cleaned potato roots heavily infected with 7–14 dpi feeding female *G. pallida* were lightly macerated using a bench top blender, and soaked in 10% formaldehyde for 3 days at room temperature. Fixed nematodes were collected by additional blending and subsequent sucrose gradient centrifugation (40% w/v). Feeding females were collected between 200 and 150 µm mesh sieves. The protocol was continued as described from the cutting stage.

### Root sectioning and immunolabelling

Lengths of potato root 14 days post infection with J2 of *G. pallida* were fixed in 4% paraformaldehyde in PEM buffer (50 mM PIPES, 10 mM EGTA, 10 mM MgSO_4_ pH 6.9) for 3 days at 4°C. Samples were dehydrated, resin embedded and sectioned according to Davies *et al.* [59] with the following alterations. Primary antibodies were raised to a 31 amino acid synthetic peptide VVRVARGEYENKCPAGPAGDVGPPGPPGPSG in a conserved region common to all Gp-HYP proteins predicted to have high antigenicity using [60]. The first 20 of which match with 100% identity to a consensus Gp-HYP-1 protein, the first 29 of which match with 100% identity to a consensus Gp-HYP-2 protein and the last 13 of which match with 100% identity to a consensus Gp-HYP-3 protein. Primary antibodies, or pre-immune sera, were hybridised at a dilution of 1 in 5 in 0.5% milk powder in PBS, and detected with a FITC-conjugated anti-rabbit secondary antibody at a dilution of 1 in 100. Plant cell walls were stained using Calcoflour-White at 1 mg/ml. Antibody specificity was tested against protein expressed in, and extracted from, the heterologous *E. coli* system (Supporting information S1).

### *In planta* RNA interference

An inverted repeat (IR) construct was generated using an entire *Gp-hyp-1* coding region. To clone in forward direction the oligonucleotide primers CTCGAG ATGGTCGGCAACAATTTG and GGTACC TTAATATTTGCATTCGCAAGC introduced a 5′ Xho I and 3′ Kpn I site respectively. To clone in the inverted direction the oligonucleotide primers TCTAGA ATGGTCGGCAACAATTTG and AAGCTT TTAATATTTGCATTCGCAAGC introduced a 5′ Xba I and a 3′ Hind III site respectively. Correct amplification was confirmed by sequencing. Sequences with no errors were cloned into the vector pHannibal [61] under the control of a CaMV 35S promoter and OCS terminator using the relevant restriction enzymes. The entire construct from promoter to terminator was cloned into the plant binary vector pART27 [62] using Sac I and Spe I. Four hundred overlapping 21 nucleotide fragments could hypothetically be generated from the IR construct. These were compared by alignment with the *Gp-hyp*-2 and -3 consensus transcripts. 26 individual 21 nucleotide fragments matched the *Gp-hyp*-2 consensus sequence with 100% identity while 10 matched to the *Gp-hyp*-3 consensus sequence. As a control, a full length GFP inverted repeat was created as above using the oligonucleotide primers CTCGAG ATGAGTAAAGGAGAAGAACTTTTC and GGTACC CTATTTGTATAGTTCATCCATGCC for the forward direction and TCTAGA ATGAGTAAAGGAGAAGAACTTTTC and AAGCTT CTATTTGTATAGTTCATCCATGCC for the reverse direction.

A single colony of *Agrobacterium rhizogenes* strain R1000 containing the relevant pART27 IR construct was incubated in 5 ml liquid Luria Bertani medium overnight at 28°C. Potato hairy root transformation was carried out by incubating 1 cm squares of *Solanum tuberosum* (‘Desirée’) leaf material in liquid MS20 medium (4.3 g/l Murashige and Skoog (with vitamins), 20 g/l sucrose, pH 5.3–5.6) containing 100 µl of *Agrobacterium* culture, for 3 days at room temperature. Leaf squares were dried on filter paper and placed on MS20 agar plates (2.4 g/l agar), containing 50 µg/ml kanamycin and 400 µg/ml cefotaxime. Roots originating from different locations on each leaf square were considered individual transformation events, and were removed and cultured on MS20 agar plates containing 50 µg/ml kanamycin. Inverted repeat construct expression was confirmed by RNA extraction (RNeasy Plant Mini Kit, Qiagen), cDNA synthesis (SuperScript II Reverse Transcriptase, Invitrogen), and PCR using the inverted repeat specific oligonucleotide primers ACGGAGGTTATGACGAG and GCTTGCGAATGCAAATATTAA.

Hatched J2 of *G. pallida* were sterilised for 20 minutes in an appropriate volume of hexadecyltrimethylammonium bromide (CTAB, 0.5 mg/ml – Sigma) containing 0.1% v/v chlorhexidine digluconate (Sigma) and 0.01% v/v Tween-20, followed by three washes in sterile tap water. J2s were suspended at a concentration of approximately 1 nematode/µl and 30 µl of suspension was pipetted onto each infection point. Three infection points were used per hairy root plate and 10 plates were used per line, with three independent lines for the *Gp-hyp*-IR, one line for GFP control, and one line for empty vector control. Two weeks after infection roots were stained by soaking in 1% sodium hypochlorite for 5 minutes, washing with tap water for 1.5 minutes 3 times, followed by boiling in 1× acid fuchsin stain for 2 minutes. Total nematode numbers per root system were counted.

### Bioinformatic analyses

Analysis of tandem repeats was carried out on genomic clones of *Gp-hyp-1 -2* and -*3* genes. For each subfamily, the middle region of the protein (between the two introns) was translated so that frame would be consistent with a cDNA clone. The software XSTREAM (http://jimcooperlab.mcdb.ucsb.edu/xstream/2013-8-8) was used to analyse the tandem repeats present for each subfamily. Putative signal peptides and transmembrane domains were predicted using SignalP v 4.1 (http://www.cbs.dtu.dk/services/SignalP/) and TMHMM v 2.0 (http://www.cbs.dtu.dk/services/TMHMM-2.0/), from the Centre for Biological Sequence analysis [63].

### Accession numbers

HYP effectors present in the *G. pallida* genome assembly (GPLIN_001208400, GPLIN_001025300, GPLIN_001135100, GPLIN_000907700) available at:

ftp://ftp.sanger.ac.uk/pub/pathogens/Globodera/pallida/Gene_Predictions/

Unique genomic sequences for all amplified, cloned *G. pallida* HYP effectors are available at GenBank under accession numbers KM206198 to KM206272.

## Supporting Information

Figure S1 **Expression of HYP-effectors throughout the life-cycle.** All *Gp-hyp* genes present in the genome assembly are specifically and highly up regulated throughout the biotrophic phases of the life cycle only. For each gene present in the genome assembly, statistically significant p values range from 1.68*10^−3^ to 5.08*10^−9^.
(TIF)

Figure S2 **Gp-HYP antibody control.** Equal quantities of whole nematode protein extract and plant root protein extract are electrophoresed alongside a very low concentration of Gp-HYP 2 purified protein. A) Gp-HYP antibody specifically detects a Gp-HYP 2 protein expressed and purified from bacteria. B) Gp-HYP antisera detect a range of proteins from a total nematode extract that are not detected using the same concentrations of pre-immune control. Neither Gp-HYP antisera nor pre-immune control can detect plant proteins at these concentrations.
(TIF)

Figure S3 **Non-canonical apparent splice events.** A single RNA species was synthesised *in vitro*. cDNA was reverse transcribed from this RNA followed by PCR analysis. A) For subfamily-1, multiple amplification products can be seen resulting from PCR on cDNA produced with either SuperScript II or Maxima RT. No such banding pattern is observed for PCR on a single gene from plasmid DNA. B) Similar additional banding patterns can be seen for subfamily-3. C) No such band pattern can be seen for a non-tandem repeat containing control GpCys. D) Denaturing RNA gel confirms a single RNA species in each case. Additional amplification products are therefore introduced as a result of the reverse transcriptase enzymes.
(TIF)

Supporting Information S1 **HYP effector protein expression, extraction and antibody specificity.**
(DOCX)

Supporting Information S2 **Non-canonical** ***in vitro*** **apparent splicing of HYP effectors as a result of reverse transcriptase template switching activity.**
(DOCX)

Table S1 **HYP effector cloning primers and annealing temperatures.**
(XLSX)

Table S2 **Summary of domains and tandem repeat motifs in deduced amino acid sequences of** ***G. pallida*** **unique genomic HYP effector sequences.**
(XLSX)
