# Supplementary figures and images for: Identification and Characterisation of a Hyper-Variable Apoplastic Effector Gene Family of the Potato Cyst Nematodes
Source: PLoS Pathog. 2014 Sep 25;10(9):e1004391. doi: 10.1371/journal.ppat.1004391 (PMC4177990; doi:10.1371/journal.ppat.1004391)

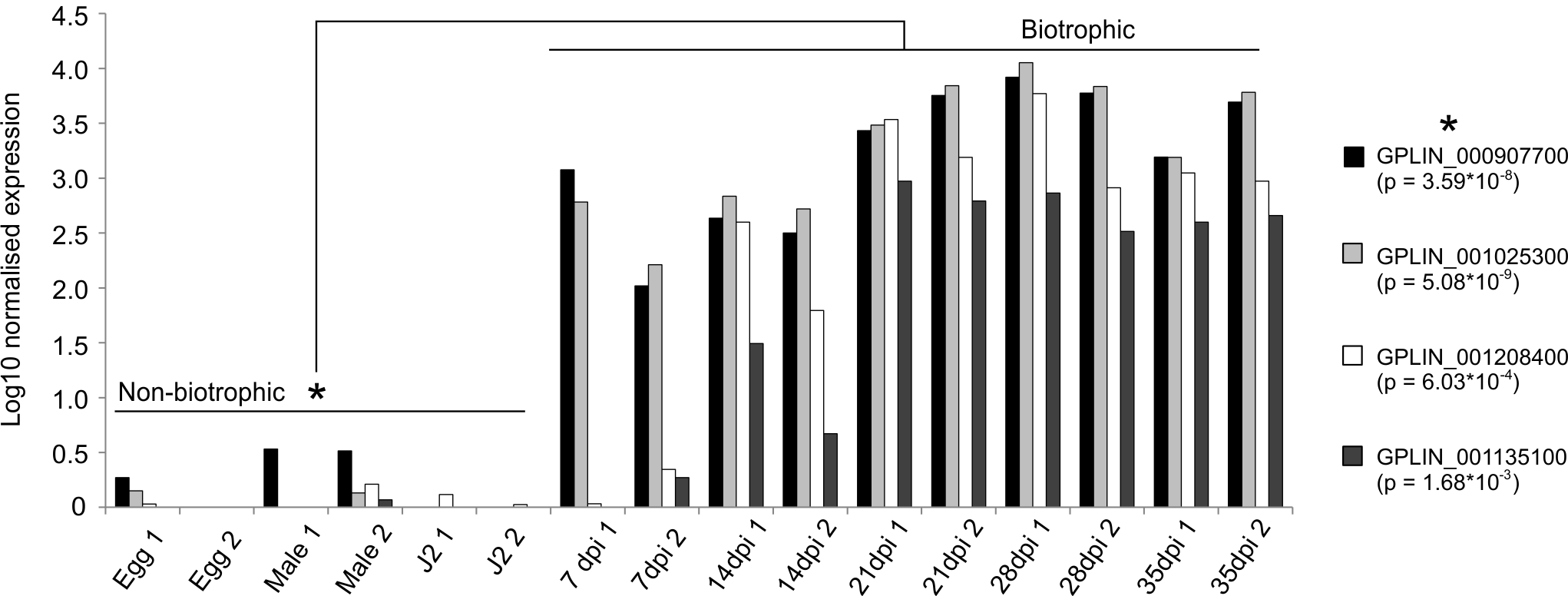

Supplement: Figure S1 — Expression of HYP-effectors throughout the life-cycle. All Gp-hyp genes present in the genome assembly are specifically and highly up regulated throughout the biotrophic phases of the life cycle only. For each gene present in the genome assembly, statistically significant p values range from 1.68*10−3 to 5.08*10−9. (TIF) [file ppat.1004391.s001.tif]

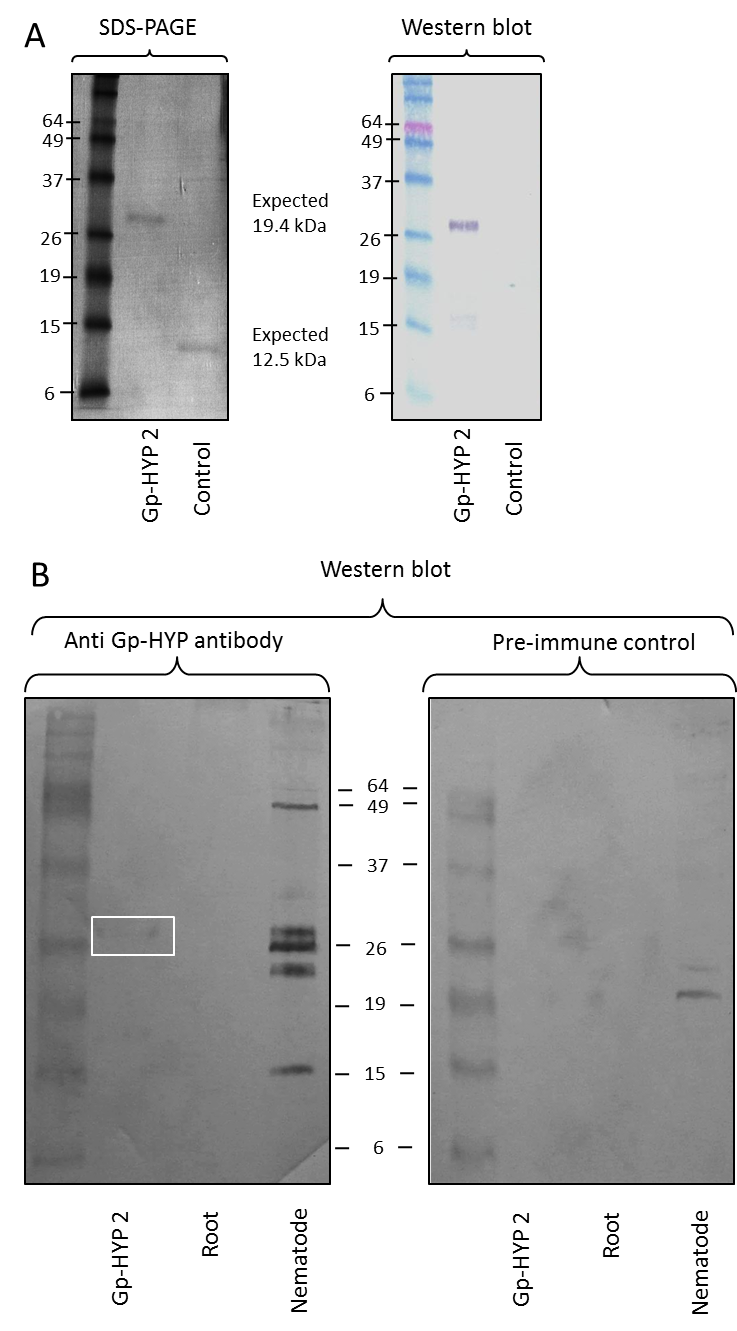

Supplement: Figure S2 — Gp-HYP antibody control. Equal quantities of whole nematode protein extract and plant root protein extract are electrophoresed alongside a very low concentration of Gp-HYP 2 purified protein. A) Gp-HYP antibody specifically detects a Gp-HYP 2 protein expressed and purified from bacteria. B) Gp-HYP antisera detect a range of proteins from a total nematode extract that are not detected using the same concentrations of pre-immune control. Neither Gp-HYP antisera nor pre-immune control can detect plant proteins at these concentrations. (TIF) [file ppat.1004391.s002.tif]
