# Supplementary material for: Identification and Characterisation of a Hyper-Variable Apoplastic Effector Gene Family of the Potato Cyst Nematodes
Source: PLoS Pathog. 2014 Sep 25;10(9):e1004391. doi: 10.1371/journal.ppat.1004391 (PMC4177990; doi:10.1371/journal.ppat.1004391)
Supplement: Supporting Information S1 — HYP effector protein expression, extraction and antibody specificity. (DOCX) [file ppat.1004391.s004.docx]

**Supporting information S1 - Protein expression, extraction and western blot**

Primers were designed to amplify the coding region, after the predicted signal peptide cleavage site, of *Gp-hyp*-2 and a control effector with the addition of the relevant restriction enzyme sites for cloning. In each case the PCR was carried out as described on plasmid DNA, as a result subfamily specific primers were not required. *Gp-hyp*-2 was amplified using the oligonucleotide primers CATATGGGCTGCAAGCCAGGACC and GGATCCTTAATATTTGCATTCACAAGCC to incorporate 5’ Nde I and 3’ Bam HI restriction enzyme sites respectively. The control gene was amplified using the oligonucleotide primers CATATGCTTATTTCCGCCTTATTTTTGG and GGATCCTCACCAGCCTCCACCG to incorporate 5’ Nde I and 3’ Bam HI restriction enzyme sites respectively. The amplicons were cloned into pGEM-T EASY by TA cloning and correct sequences confirmed by sequencing. 2 μg of plasmid containing the relevant gene fragment was digested with Nde I and Bam HI. The released gene fragments were ligated into the bacterial expression vector pPET28b vector, digested in a similar manner. Expression constructs containing the gene of interest were transformed into a chemically competent expression strain of *Escherichia coli*, BL21 DE3-RIL. A single colony containing the relevant plasmid was cultured in 5 ml Luria-Bertani (LB) medium at 37 ^o^C with shaking until A_600_ =0.5–0.8. Protein expression was induced by addition of IPTG to a final concentration of 1 mM, and incubation for 3 hours at 28 ^o^C. HIS-tagged protein was extracted under denaturing conditions by resuspending cell pellets in 8 M Urea, centrifugation at 12,000 *g* for 5 minutes, and incubating the supernatant with Ni-NTA resin (Qiagen) for 30 minutes at room temperature. Unbound protein was removed from the beads by centrifugation and wash steps and bound proteins were cleaved from the resin by addition of EDTA. Purified protein was separated from Ni-NTA resin by centrifugation and analysed by SDS-PAGE.

Protein was extracted from either frozen nematode or plant tissue by thoroughly grinding with a cooled micro-pestle in liquid nitrogen. The resultant powder was resuspended in an appropriate volume of phosphate buffered saline (PBS: 137 mM NaCl, 2.7 mM KCl, 8 mM Na_2_HPO_4_, 1.5 mM KH_2_PO_4_, pH 7.4) with the addition of PMSF to a final concentration of 0.1 mM. The sample was centrifuged at 12,000 *g* for 3 minutes, the supernatant containing the soluble protein was removed to a new chilled microfuge tube, and the centrifugation process repeated until the supernatant was clear. The protein extract was quantified on a Nanodrop spectrophotometer ND-1000 (Thermo, Massachusetts, USA) at A_280_. Equal quantities of protein extracted from plant or nematode samples were electrophoresed on a 12 % SDS-PAGE gel for 1 hour at 200 V.

Total protein extract or purified expressed proteins immobilised on SDS-PAGE were transferred to Immobilon-P PVDF membrane (Millipore, Billerica, USA) for 1 hour at 100 V. Following transfer, the membrane was rinsed for 5 minutes in 1 x PBS. The blot was then placed into blocking solution (PBS/0.05 % Tween-20, 5 % w/v non-fat milk powder) for 1 hour at room temperature. The blocking solution was replaced with the primary antibody solution diluted 1:5000 in PBS/0.5 % Tween-20 (PBST) with 0.5 % non-fat milk powder for a minimum of 1 hour at room temperature or overnight at 4 ^o^C. The membrane was then washed 6 x 10 minutes in PBST at room temperature, prior to incubation for 1 hour at room temperature in the alkaline phosphatase-conjugated anti-rabbit antibody diluted 1:2000 in PBST with 0.5 % milk powder. The membrane was washed a further 6 x 10 minutes in PBST at room temperature. A substrate tablet (SigmaFast Tablets BCIP/NBT) was dissolved in 10 ml of water, applied to the protein side of the membrane and incubated in the dark at room temperature until bands became visible. The membranes were then washed in dH_2_O to stop the reaction and allowed to air dry.
