# Supplementary material for: Identification and Characterisation of a Hyper-Variable Apoplastic Effector Gene Family of the Potato Cyst Nematodes
Source: PLoS Pathog. 2014 Sep 25;10(9):e1004391. doi: 10.1371/journal.ppat.1004391 (PMC4177990; doi:10.1371/journal.ppat.1004391)
Supplement: Supporting Information S2 — Non-canonical in vitro apparent splicing of HYP effectors as a result of reverse transcriptase template switching activity. (DOCX) [file ppat.1004391.s005.docx]

**Supporting information S2 – Non-canonical splicing.**

Additional variation in the tandem repeat regions was noticed when cloning *Gp-hyp* sequences from cDNA compared to cloning from genomic DNA. Although the known list of genomic DNA sequences is not exhaustive, these additional variants could not be aligned with any known splice sites when comparing to cloned genomic DNA sequences. Gene splicing typically follows a well conserved CAG:GTAAGT motif at the 5’ splice site, where the splice cut will form before the underlined GT and is joined after a corresponding 3’ AG. Sequences more similar to this exon/intron boundary are more faithfully spliced than more diverged sequences. In less than 1 % of cases a GC can replace the GT with no discernable change to splicing efficiency ([Burset, et al. 2000](#_ENREF_1)). A combination of GT:AG or GC:AG splice sites could be used to explain some, but not all, of the additional variation observed in cDNA samples compared to gDNA samples. Recently it has been shown that during the cDNA synthesis process *in vitro*, RT enzymes are capable of “skipping” sections of RNA, in a homology dependent manner, to produce a truncated cDNA that resembles a typical gene splicing event ([Cocquet, et al. 2006](#_ENREF_2); [Houseley and Tollervey 2010](#_ENREF_4)). An experiment was carried out to determine if this was occurring with HYP effector sequences.

Two micrograms of template plasmid DNA containing either a single HYP effector cDNA sequence or a contronl gene without tandem repeat (GpCys) were digested with two restriction enzymes for each construct in order to ensure complete linearistion. For HYP subfamily clones NdeI and SpeI were used while KpnI and XhoI were used for the GpCys control. RNA was synthesised from 400 nanograms of cleaned linear template DNA using the MEGAscript *in vitro* transcription kit (Ambion) following the manufacturer’s instructions. The reaction was then purified using the RNeasy Plant Mini Kit (Qiagen), following the manufacturer’s instructions for “RNA cleanup”, including the optional on-column DNA digestion. Gene specific cDNA was synthesised from the purified RNA using either SuperScript II reverse transcriptase (Invitrogen) or Maxima Reverse transcriptase (Thermoscientific) according to the manufacturer’s instructions. In each case 220 ng of RNA was used, with the relevant gene specific UTR primer. For SuperScript II cDNA was synthesised at 42 ^o^C. For Maxima reverse transcriptase the optional 65 ^o^C incubation for 5 minutes was carried out, and cDNA was synthesised at 65 ^o^C. Plasmid template, synthesised RNA, and synthesised cDNA were analysed by PCR.

For *Gp-hyp-*1, PCR on cDNA synthesised with either enzyme resulted in multiple amplification products (Figure S2 A). This was not the case when amplifying from the plasmid used to generate the RNA *in vitro* (Figure S2 A). No amplification could be observed from PCR on the RNA samples. Similar banding patterns were also seen for *Gp-hyp-*3 (Figure S2 B), but not for the GpCys control (Figure S2 C). A single RNA species was confirmed by denaturing gel electrophoresis (Figure S2 D). Taken together, these results suggest that the non-canonical splicing events generated are independent of PCR, temperature, and transcriptase and result of the template switching activity of the reverse transcriptase enzyme. Cloned PCR products were sequenced to ensure the additional products were not a result of mis-priming. The template switching ability of reverse transcriptase enzymes is a remnant of their activity in viruses. Successful retroviral replication requires at least two template switching events between areas of high homology ([Gilboa, et al. 1979](#_ENREF_3)). The non-canonical splice events observed are always in the tandem repeat regions. These regions in the Gp-hyp-1 and -3 genes provide numerous areas of high homology resulting in multiple opportunities for the reverse transcriptase to switch template.

These factors made it impossible to definitively identify genuine alternative splicing events. Therefore, where possible all subsequent experiments describing the variation in HYP effectors were carried out at the DNA level.

**Supporting references:**

Burset M, Seledtsov I, Solovyev V. 2000. Analysis of canonical and non-canonical splice sites in mammalian genomes. Nucleic Acids Research 28(21):4364-4375.

Cocquet J, Chong A, Zhang G, Veitia RA. 2006. Reverse transcriptase template switching and false alternative transcripts. Genomics 88(1):127-131.

Gilboa E, Mitra SW, Goff S, Baltimore D. 1979. A detailed model of reverse transcription and tests of crucial aspects. Cell 18(1):93-100.

Houseley J, Tollervey D. 2010. Apparent non-canonical trans-splicing is generated by reverse transcriptase in vitro. Plos One 5(8):e12271.
